# Diagnostic Value of Fully Automated Artificial Intelligence Powered Coronary Artery Calcium Scoring from 18F-FDG PET/CT

**DOI:** 10.3390/diagnostics12081876

**Published:** 2022-08-03

**Authors:** Claudia Morf, Thomas Sartoretti, Antonio G. Gennari, Alexander Maurer, Stephan Skawran, Andreas A. Giannopoulos, Elisabeth Sartoretti, Moritz Schwyzer, Alessandra Curioni-Fontecedro, Catherine Gebhard, Ronny R. Buechel, Philipp A. Kaufmann, Martin W. Huellner, Michael Messerli

**Affiliations:** 1 Department of Nuclear Medicine, University Hospital Zurich, 8091 Zurich, Switzerland; claudia.morf@uzh.ch (C.M.); thomas.sartoretti@usz.ch (T.S.); gennari_antonio@libero.it (A.G.G.); alexander.maurer@usz.ch (A.M.); stephan.skawran@usz.ch (S.S.); andreas.giannopoulos@usz.ch (A.A.G.); elisabeth.sartoretti@uzh.ch (E.S.); catherine.gebhard@usz.ch (C.G.); ronny.buechel@usz.ch (R.R.B.); pak@usz.ch (P.A.K.); martin.huellner@usz.ch (M.W.H.); 2 University of Zurich, 8006 Zurich, Switzerland; 3 Institute of Diagnostic and Interventional Radiology, University Hospital Zurich, 8091 Zurich, Switzerland; moritz.schwyzer@usz.ch; 4 Department of Medical Oncology and Hematology, University Hospital Zurich, 8091 Zurich, Switzerland; alessandra.curioni@usz.ch; 5 Center for Molecular Cardiology, University of Zurich, 8006 Zurich, Switzerland

**Keywords:** artificial intelligence, coronary artery calcium scoring, coronary artery disease, deep learning, positron emission tomography

## Abstract

Objectives: The objective of this study was to assess the feasibility and accuracy of a fully automated artificial intelligence (AI) powered coronary artery calcium scoring (CACS) method on ungated CT in oncologic patients undergoing 18F-FDG PET/CT. Methods: A total of 100 oncologic patients examined between 2007 and 2015 were retrospectively included. All patients underwent 18F-FDG PET/CT and cardiac SPECT myocardial perfusion imaging (MPI) by 99mTc-tetrofosmin within 6 months. CACS was manually performed on non-contrast ECG-gated CT scans obtained from SPECT-MPI (i.e., reference standard). Additionally, CACS was performed using a cloud-based, user-independent tool (AI-CACS) on ungated CT scans from 18F-FDG-PET/CT examinations. Agatston scores from the manual CACS and AI-CACS were compared. Results: On a per-patient basis, the AI-CACS tool achieved a sensitivity and specificity of 85% and 90% for the detection of CAC. Interscore agreement of CACS between manual CACS and AI-CACS was 0.88 (95% CI: 0.827, 0.918). Interclass agreement of risk categories was 0.8 in weighted Kappa analysis, with a reclassification rate of 44% and an underestimation of one risk category by AI-CACS in 39% of cases. On a per-vessel basis, interscore agreement of CAC scores ranged from 0.716 for the circumflex artery to 0.863 for the left anterior descending artery. Conclusions: Fully automated AI-CACS as performed on non-contrast free-breathing, ungated CT scans from 18F-FDG-PET/CT examinations is feasible and provides an acceptable to good estimation of CAC burden. CAC load on ungated CT is, however, generally underestimated by AI-CACS, which should be taken into account when interpreting imaging findings.

## 1. Introduction

Hybrid 18F-fluorodeoxyglucose positron emission tomography (18F-FDG PET) with computed tomography (CT) has evolved as an important imaging modality for staging and restaging of oncological patients [1]. Clinical 18F-FDG PET/CT examinations consist of a PET scan and a non-contrast, free-breathing, ungated CT. The CT is used for (*a*) PET attenuation correction but (*b*) also includes relevant morphological information regarding disease/tumor extent. Even though the appropriate oncological diagnosis and treatment planning is the primary concern in cancer patients, relevant comorbidities should not be underestimated and should ideally be described in the imaging report. Indeed, a recent population-based study including more than 3 million cancer patients indicated that the highest number of cardiovascular deaths occurred in the first year following initial cancer [2].

Coronary artery calcium (CAC) is an important biomarker in patients with coronary heart disease (CHD) [3,4]. Increased CAC scores are strongly associated with cardiovascular mortality and all-cause mortality [3]. Hybrid 18F-FDG PET/CT examinations are generally not suited for the comprehensive evaluation of CAC or CHD, as the CT scan is neither acquired nor reconstructed with the appropriate scan parameters as recommended for dedicated cardiac CT calcium scans. Therein, the lack of ECG-gating, the use of iterative reconstruction algorithms, and the specific choice of field of view, slice thickness, kernel, and tube voltage can be challenging [5,6,7]. Nonetheless, an opportunistic screening resulting in the rough estimation of the coronary disease burden by means of CAC would be highly desirable; indeed, this was recommended in a recent consensus statement of the British Societies of Cardiovascular Imaging/Cardiac Computed Tomography and Thoracic Imaging [8]. Optimally, this assessment (i.e., CAC scoring, CACS) should be performed fully automatically so that the physician can continue to focus on the oncological workup of the scan. With recent advances in the field of artificial intelligence (AI) for medical imaging [9,10,11,12], deep-learning (DL) powered calcium scoring tools have been developed that allow for the quantitative assessment of CAC in a fully automated manner [11,12,13,14].

Given the considerations outlined above, these tools would be suited for the opportunistic assessment of CAC in patients undergoing oncologic 18F-FDG PET/CT examinations, as quantitative CAC scores are provided without having to perform CAC scoring manually.

In this study, we sought to test the feasibility of such an approach. Specifically, we assessed the quantitative accuracy of an AI-powered CACS tool in estimating CAC from CT scans acquired during oncologic 18F-FDG PET/CT examinations using manual CACS measurements from a dedicated cardiac imaging workup as the standard of reference.

## 2. Material and Methods

### 2.1. Study Population and Study Design

**Figure 1:**
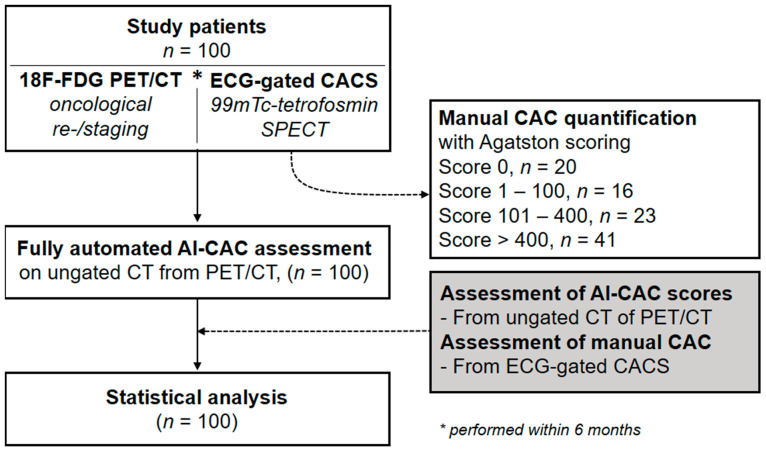
Flow chart of study.

This study was approved by the local ethics committee (BASEC No. 2017- 01112; Kantonale Ethikkommission, Kanton Zürich, Switzerland; secondary approval on 07.04.2021), and the need for informed consent was waived due to the retrospective nature of the study. The study population was partly shared in previous studies [1,15]. Our study population was selected from a retrospective cohort study of consecutive patients undergoing (*a*) a whole-body 18F-FDG-PET/CT for malignant disorders at the University Hospital of Zurich between November 2007 and February 2015, and (*b*) 1-day stress/rest (regadenoson, adenosine, dobutamine, or exercise) myocardial perfusion imaging by 99mTc-tetrofosmin single-photon emission computed tomography (SPECT-MPI) including non-contrast, ECG-gated CT for attenuation correction within 6 months of 18F-FDG-PET/CT imaging to evaluate known or suspected CAD (Figure 1). CAC scoring was performed manually on the dedicated non-contrast ECG-gated CT scans (120 kV, reconstructed with weighted filtered back projection, a slice thickness of 3 mm, and an increment of 1.5 mm) as obtained during myocardial perfusion imaging by two experienced physicians in consensus (i.e., reference standard) using a dedicated software program (Smartscore, GE Healthcare, Milwaukee, WI, USA) [16]. Out of 100 selected patients, 20 patients were identified with a CAC score of 0, 16 patients with a score of 1–100, 23 patients with a score of 101–400, and 41 patients with a score of >400. An overview of the patient demographics is provided in Table 1. Next, scores from manual CACS as performed on dedicated non-contrast ECG-gated CT scans (i.e., reference standard) were compared to scores from AI-CACS as performed on CT scans from 18F-FDG-PET/CT imaging (see: Section 2.3).

As a preliminary proof-of-concept, we also tested whether manual CACS and AI-CACS can theoretically be performed on all datasets (i.e., non-contrast ECG-gated CT scans from SPECT-MPI and CT scans from 18F-FDG-PET/CT imaging) effortlessly. Thus, manual CACS and AI-CACS was performed on all datasets of 15 patients. These data are provided solely in the Supplementary Material.

### 2.2. Whole Body 18F-FDG PET/CT Including Ungated CT

Patients underwent PET/CT imaging from skull to pelvis one hour after injection of 18F-FDG (including a non-contrast, free-breathing, ungated CT scan). Images were acquired in 3D mode on a Discovery VCT or Discovery RX scanner (GE-Healthcare, Milwaukee, WI, USA). PET/CT and CT images were merged and analyzed using Advantage Window Volume Viewer software (GE-Healthcare, Milwaukee, WI, USA) [1].

### 2.3. Fully Automated AI-CAC Scoring

AI-CACS was performed with a fully automated deep-learning based CAC scoring tool (AVIEW CAC, Coreline Soft, access via https://cloud.corelinesoft.eu/login accessed on 1 May 2022). In brief, the software was developed based on a 3-dimensional U-net architecture using non-enhanced cardiac CT scans acquired from multiple vendors and scanners. A more detailed description of the network architecture and the algorithm, including information on initial training datasets and validation procedures, can be found elsewhere [17,18]. No training data were included in this current study [14,17]. Thus, this study represents an external validation and test of the AI-CACS algorithm under clinically realistic conditions. Initially, the non-contrast, free-breathing, ungated CT scans from the 18F-FDG PET/CT examination were postprocessed in the hospital’s PACS system by cropping the image series. Specifically, a second dataset encompassing all images from the lung apex to the lung base was generated for each patient. This anonymized image series was then transferred to the AI tool. Fully automated CACS was then performed without any further user input. The results from CACS were then summarized in a report generated by the AI tool.

### 2.4. Statistical Analysis

The data were initially presented with descriptive statistics. Diagnostic accuracy parameters were computed to quantify the AI tool’s ability to correctly identify coronary calcium relative to the reference standard. Quantitative CAC scores between the AI tool and the reference standard were compared by means of intraclass correlation coefficient (ICC) analysis, linear regression modelling, and Bland–Altman analysis. Interclass agreement of CAC risk category classes was quantified by means of weighted Kappa analysis. For ICC and weighted Kappa analysis, the following scale was considered for results interpretation: poor (ICC, *k* < 0.20), fair (ICC, *k* = 0.21–0.40), moderate (ICC, *k* = 0.41–0.60), good (ICC, *k* = 0.61–0.80), and excellent (ICC, *k* = 0.81–1.00) agreement [19]. All statistical analyses were performed in the R programming language (https://www.r-project.org accessed on 1 May 2022).

## 3. Results

A total of 100 patients who underwent both whole-body 18F-FDG PET/CT and SPECT-MPI (including a dedicated ECG-gated CACS) within a 6-month period were enrolled. In all patients, the AI-CACS tool successfully managed to process the dataset (i.e., ungated low dose CT).

### 3.1. Diagnostic Accuracy of AI-CACS for the Detection of Coronary Calcifications

The sensitivity of the AI-CACS tool for the detection of coronary calcifications analyzed per-patient was 85.0%, and analyzed per-coronary artery analysis 74.5% (left main, LM), 82.0% (left anterior descending, LAD), 64.2% (left circumflexus, LCX), and 61.7% (right coronary artery, RCA), respectively. Further results of the per-patient and per-coronary artery diagnostic performance of AI-CACS are presented in Table 2.

### 3.2. Quantitative Agreement of AI-CACS with Manual CACS

**Figure 2:**
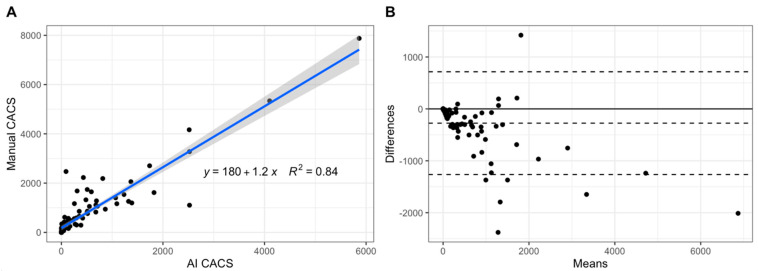
Scatter plot depicting a linear regression model between CAC scores from AI-CACS and manual CACS (**A**) as well as a Bland–Altman plot showing the relationship between CAC scores from AI-CACS and manual CACS (**B**).

Interscore agreement (i.e., ICC) of CAC scores between the AI tool and manual measurements as the reference standard was 0.88 (95% CI: 0.827, 0.918). The linear regression model between CAC scores of the AI tool and the reference standard (Figure 2) revealed an R^2^ of 0.84, an intercept of 180, and a slope of 1.2. Bland–Altman analysis showed a bias of 274.8 and a lower and upper limit agreement of −714.9 and 1264.5, respectively (see Figure 2). On a per-vessel basis, interscore agreement was 0.761 (95% CI: 0.664, 0.831) for LM, 0.863 (95% CI: 0.803, 0.906) for LAD, 0.716 (95% CI: 0.605, 0.799) for CX, and 0.812 (95% CI: 0.733, 0.869) for RCA.

In terms of risk category classes (Table 3), a weighted Kappa score of 0.800 was found for the interclass agreement between the AI tool and the reference standard. Reclassification of risk category occurred in 44 cases (44%), of which there was shifting by one category in 39 cases (89%) and by two categories in 5 cases (11%). In 42 cases (42%), the AI tool underestimated the risk category, whereas in 2 cases (2%), the AI tool overestimated the CAC burden (risk class 1 instead of risk class 0).

**Figure 3:**
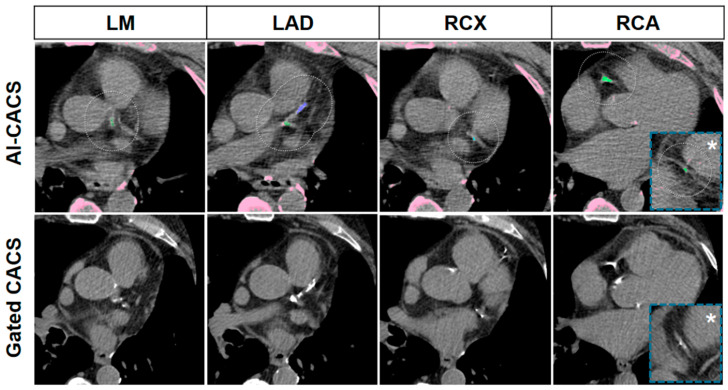
Representative CT images of a 69-year-old man with a body mass index of 26.3 kg/m^2^ with severe coronary artery calcifications. Images from ungated CT from a PET/CT performed for restaging of a rectal adenocarcinoma are presented in the upper row, and dedicated gated CAC CT from myocardial perfusion single photon emission computed tomography performed 99 days later are presented in the lower row. Coronary calcifications in the left main (LM), left anterior descending (LAD), ramus circumflexus (RCX), and right coronary artery (RCA) including the distal segment (asterisk), were correctly marked by the AI-CACS tool resulting in a score of 865. The score from the dedicated CAC scan was 942.

**Figure 4:**
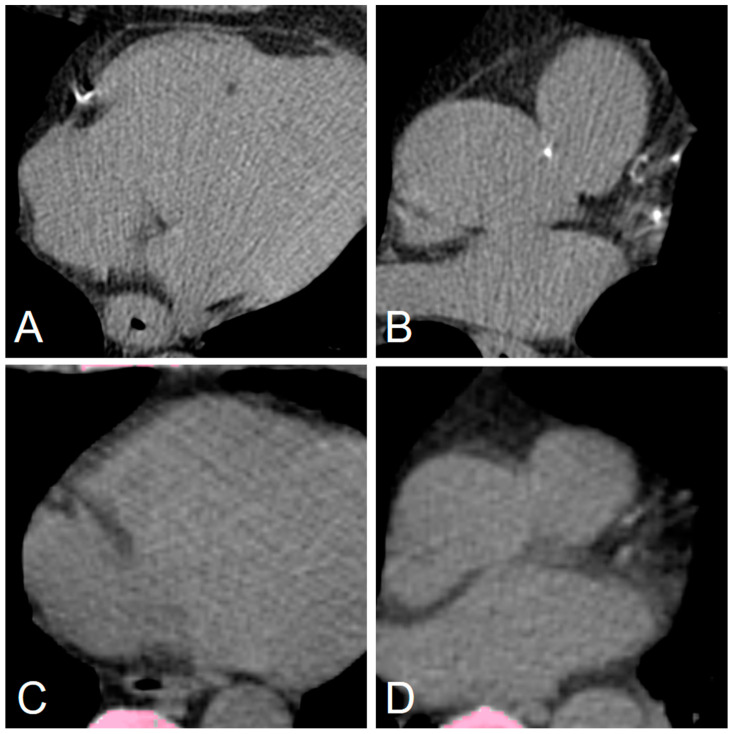
Representative false negative markings from the AI-CACS tool on ungated CT from PET/CT in a 73-year-old man undergoing PET/CT. Dedicated gated coronary calcium scan showed small coronary calcifications in the right coronary artery (**A**) and left anterior descending as well as left circumflex artery (**B**). However, the calcifications are not depicted on ungated low dose PET/CT (**C**,**D**) performed for staging of lung cancer.

**Figure 5:**
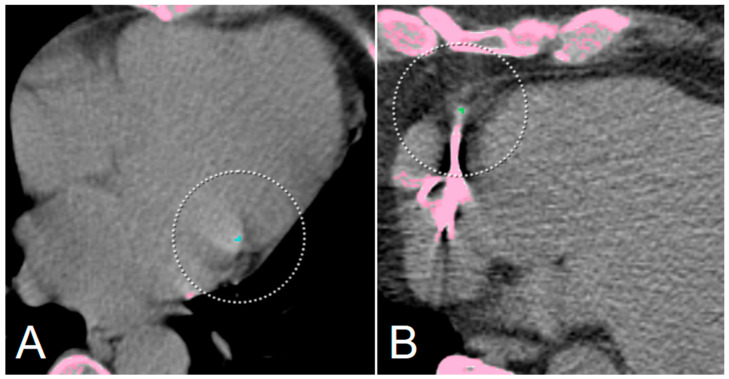
Two representative false-positive ratings from the AI-CACS tool on ungated CT from PET/CT. No coronary calcifications are present in a 66-year-old woman undergoing PET/CT for staging of gastric cancer (**A**), however, small calcifications of mitral valve were falsely marked as ramus circumflexus CAC. In another 66-year-old male patient (**B**), no CAC was found in dedicated gated CAC scan, however, small areas of increased density due to image noise adjacent to a pacemaker electrode were marked as calcification. Note: Pink areas are highlighing dense areas in the CT image (i.e., bone or calcification), with the circles indicating coronary artery calcification.

Representative cases of the AI-CACS tool correctly identifying the coronary calcium burden in a patient are presented in Figure 3. Further examples presenting false negative findings as well as the two patients of the study cohort that were falsely classified to have an Agatston Score > 0 (i.e., false positive findings) are presented in Figure 4 and Figure 5, respectively.

## 4. Discussion

In this retrospective study, we aimed to assess the value of a fully automated AI tool to accurately quantify CAC in patients undergoing non-contrast free-breathing ungated CT as part of an oncologic 18F-FDG PET/CT examination.

Our data indicate that the AI tool manages to detect and quantify CAC with acceptable to good accuracy without requiring any user input. However, AI-CACS from ungated CT generally underestimates CAC burden, which should be kept in mind. Nonetheless, this study further provides evidence that CAC scores can be extracted effortlessly from various types of CT scans, thus potentially expanding the diagnostic value and impact of a given examination.

Following a recent consensus statement from the British Societies of Cardiovascular Imaging/Cardiac Computed Tomography and Thoracic Imaging, physicians are urged to report incidental coronary calcifications on all CT scans covering the chest, as CAC is an important marker of CAD in both symptomatic and asymptomatic patients [8]. Specifically, CAC is associated with a poorer prognosis in various patient groups, including cancer patients. Notably, a sub-analysis of the National Lung Screening Trial showed that CAC scores of >100 were associated with a four to sevenfold increase in mortality risk as compared to patients without CAC [20]. For CAC grading, the authors recommend using a semi-quantitative ordinal scoring system instead of the conventional quantitative Agatston scoring system. While the authors acknowledge that the Agatston scoring system represents the gold standard assessment for CAC, they point out that the additional time effort and use of dedicated software may prevent physicians from implementing and performing CACS on non-dedicated CT scans, as in the case of PET/CT imaging [8].

In the current study, we present a viable approach that enables physicians to extract quantitative Agatston scores from ungated CT scans as acquired for attenuation correction of oncologic PET scans. This AI tool runs fully automatically without any further user input and generates a detailed CACS report that can directly be sent to the user or to the institutions’ PACS. Notably, the tool has previously been validated by Vonder et al., who tested the tool’s performance relative to manual CACS measurements in a cohort of 997 patients who had undergone a dedicated cardiac CT protocol, including calcium scans, as part of a cardiovascular screening program. The authors found an interscore agreement of 0.958 and an interclass agreement of 0.96 for risk categories, thus confirming the AI tool’s ability to perform CACS accurately on dedicated cardiac calcium CT scans [14].

In contrast, we found an interscore agreement of 0.88 and an interclass agreement of 0.800 for risk categories. In this regard, it should be noted that dedicated calcium scans are performed in breath-hold and with ECG-gating. This is not the case for the CT acquired during PET/CT examinations and may therefore significantly impact CAC quantification accuracy. Specifically, it has been shown that the calcium load can be underestimated on ungated CT scans [21,22,23,24]. Furthermore, it should be noted that the acquisition and reconstruction parameters of the CT scan from PET/CT imaging may differ from those recommended for a dedicated cardiac calcium scan. For example, the latter should be performed at 120 kV and should be reconstructed with weighted filtered back projection [5,7].

Despite these differences and challenges, our AI tool achieved an acceptable to good performance in detecting and quantifying CAC. Specifically, although reclassification of risk categories frequently occurred (44% of cases), risk categories nearly always (i.e., 89%) shifted by only one category. Furthermore, in nearly all cases, the risk category was underestimated by the AI tool, which may partially be due to the inherent limitations of an ungated CT scan for CAC detection. When interpreting CAC scores as obtained from AI-CACS on CT scans from PET/CT, this should be kept in mind nonetheless.

Finally, we would like to emphasize that we did not further optimize the AI tool prior to study onset by performing any specific or further training on our dataset. Thus, the data used in the current study represent a true validation set. In this regard, it should be noted that the performance of the AI tool may be further improved in the future by training the algorithm with further study/institution specific data. Importantly, we suspect that additional training with ungated CT scans may prove valuable in improving the tools accuracy.

Our study has the following limitations: First, this was a retrospective single-center study with a limited number of subjects. Nonetheless, despite the specific and selective inclusion criteria, we achieved a sample size comparable to that of similar studies [18,25].

In terms of study subject selection, it should be acknowledged that the results inherently depend on the examined patient cohort. Here, we used a unique and heterogeneous patient cohort of oncologic patients with scans ranging back to 2007. Incidentally, the AI tool may provide even better results when using more recent scans and scans from a more homogenous patient cohort (performed on more modern scanners). Second, we did not perform manual CACS or semi-quantitative visual grading of CAC on the CT scans from PET/CT imaging. This would have allowed us to better quantify the measurement inaccuracy of the AI tool itself. This should be investigated in future studies. Third, as a reference standard, we used manual CACS scores from a dedicated cardiac SPECT-MPI examination performed within 6 months of the oncologic PET/CT. CAC scores are not expected to change within this time frame; nevertheless, it should be acknowledged that minor changes may have occurred, thus potentially introducing a bias.

In conclusion, our study indicates that an AI tool enables fully automatic and effortless calcium scoring on non-contrast free-breathing, ungated CT scans from 18F-FDG-PET/CT examinations, thereby providing an acceptable to good estimation of the CAC burden. CAC load on ungated CT is, however, generally underestimated by AI-CACS, which should be taken into account when interpreting imaging findings. Nonetheless, our findings provide evidence that physicians can effortlessly achieve an acceptable to good estimation of the CAC burden from oncologic 18F-FDG PET/CT examinations, thus potentially enabling an opportunistic screening of CAD and allowing for the further expansion of the diagnostic spectrum and value of the imaging modality.

## Figures and Tables

**Table 1:** Demographics of study patients (*n* = 100).

Female/Male	34/66
Age, years	66 ± 11.4 (32–91)
Weight, kg	74.2 ± 16.5 (46.8–140.0)
Height, cm	169.8 ± 9.6 (135–200)
BMI, kg/m^2^	25.6 ± 5.2 (17.2–47.3)
Primary Tumor, n (%)	
Head and neck cancer	11%
Lung or pleural cancer	12%
Rectal or colon cancer	13%
Esophageal cancer	20%
Liver tumor	5%
Breast cancer	7%
Pancreatic and biliary cancer	8%
Lymphoma	4%
Others	20%

BMI: body mass index, presented as % and mean ± SD (range).

**Table 2:** Diagnostic performance of AI-CACS with manual CACS as reference. Data are shown per patient and per coronary artery.

	Per Patient	Per Coronary Artery			
	All Patients	LM	LAD	RCX	RCA
Sensitivity	85.0% (77.2–92.8%)	74.5% (62.5–86.5%)	82.0% (73.4–91.0%)	64.2% (51.2–77.1%)	61.7% (49.4–74.0%)
Specificity	90.0% (76.9–100%)	79.6% (68.3–90.9%)	100% (100–100%)	95.7% (90.0–100%)	95.0% (88.2–100%)
Diagnostic accuracy	86.0% (77.6–92.1%)	77.0% (67.5–84.8%)	87.0% (78.8–92.9%)	79.0% (69.7–86.5%)	75.0% (65.3–83.1%)
PPV	97.1% (93.2–100%)	79.2% (67.7–90.7%)	100% (100–100%)	94.4% (87.0–100%)	94.9% (88.0–100%)
NPV	60.0% (42.5–77.5%)	75.0% (63.2–86.8%)	67.5% (53.0–82.0%)	70.3% (59.1–81.5%)	62.3% (50.1–74.4%)
Total, n	100	100	100	100	100
True positive, n	68	38	60	34	37
False negative, n	12	13	13	19	23
True negative, n	18	39	27	45	38
False positive, n	2	10	0	2	2

LM left main, LAD left anterior descending, RCX ramus circumflex, RCA right coronary artery, PPV positive predictive value, NPV negative predictive value. Data are presented as sensitivity, specificity, diagnostic accuracy, PPV, and NPV % (95% confidence interval).

**Table 3:** Confusion matrices of risk categories between AI-CACS and manual CACS. Weighted Kappa values were 0.8.

Manual CACS	AI-CACS				Total	Underestimation ^a^	Overestimation ^a^	Concordance ^a^
	0	1–100	101–400	>400				
0	18	2	0	0	20	-	2 (10.0%)	18 (90.0%)
1–100	11	5	0	0	16	11 (68.8%)	0	5 (31.2%)
101–400	1	17	5	0	23	18 (78.3%)	0	5 (21.7%)
>400	0	4	9	28	41	13 (31.7%)	-	28 (68.3%)

CACS coronary artery calcium scoring; ^a^ i.e., manual coronary artery calcium scoring as reference.

## Data Availability

Data can be made available upon reasonable request to the corresponding author.

## Supplementary Materials

The following supporting information can be downloaded at: https://www.mdpi.com/article/10.3390/diagnostics12081876/s1, Table S1: Manual CACS and AI-CACS on ungated CT from PET/CT and dedicated gated CAC CT from myocardial SPECT/CT.

## Abbreviations

AI	Artificial intelligence
CAC	Coronary artery calcium
CACS	Coronary artery calcium scoring
CHD	Coronary heart disease
CT	Computed tomography
DL	Deep-learning
F18-FDG	F18-Fluorodeoxyglucose
MPI	Myocardial perfusion imaging
PET	Positron emission tomography
SPECT	Single-photon emission computed tomography
SUV_max_	Maximum standardized uptake value
